# The Impact of Cancer-Associated Fibroblasts on the Biology and Progression of Colorectal Carcinomas

**DOI:** 10.3390/genes15020209

**Published:** 2024-02-06

**Authors:** Larissa Maria Henrich, Kristina Greimelmaier, Michael Wessolly, Nick Alexander Klopp, Elena Mairinger, Yvonne Krause, Sophia Berger, Jeremias Wohlschlaeger, Hans-Ulrich Schildhaus, Hideo Andreas Baba, Fabian Dominik Mairinger, Sabrina Borchert

**Affiliations:** 1 Institute of Pathology, University Hospital Essen, University of Duisburg Essen, 45147 Essen, Germany; larissa.henrich@rlk.uk-essen.de (L.M.H.); michael.wessolly@uk-essen.de (M.W.); nick.klopp@gmx.de (N.A.K.); elena.mairinger@gmail.com (E.M.); hideo.baba@uk-essen.de (H.A.B.); sabrina.borchert@rlk.uk-essen.de (S.B.); 2 Department of Pathology, Diakonissenkrankenhaus Flensburg, 24939 Flensburg, Germanywohlschlaegerje@diako.de (J.W.); 3 Targos-A Discovery Life Sciences Company, Germaniastraße 7, 34119 Kassel, Germany; hans-ulrich.schildhaus@dls.com; 4 Institute of Pathology Nordhessen, Germaniastraße 7, 34119 Kassel, Germany

**Keywords:** colorectal cancer, CRC, tumour microenvironment, cancer-associated fibroblasts, fibroblast activating protein, FAP

## Abstract

(1) Colorectal cancer (CRC) is a leading cause of cancer-related deaths globally. Cancer-associated fibroblasts (CAFs) are major components of CRC’s tumour microenvironment (TME), but their biological background and interplay with the TME remain poorly understood. This study investigates CAF biology and its impact on CRC progression. (2) The cohort comprises 155 cases, including CRC, with diverse localizations, adenomas, inflammations, and controls. Digital gene expression analysis examines genes associated with signalling pathways (MAPK, PI3K/Akt, TGF-β, WNT, p53), while next-generation sequencing (NGS) determines CRC mutational profiles. Immunohistochemical FAP scoring assesses CAF density and activity. (3) FAP expression is found in 81 of 150 samples, prevalent in CRC (98.4%), adenomas (27.5%), and inflammatory disease (38.9%). Several key genes show significant associations with FAP-positive fibroblasts. Gene set enrichment analysis (GSEA) highlights PI3K and MAPK pathway enrichment alongside the activation of immune response pathways like natural killer (NK)-cell-mediated cytotoxicity via CAFs. (4) The findings suggest an interplay between CAFs and cancer cells, influencing growth, invasiveness, angiogenesis, and immunogenicity. Notably, TGF-β, CDKs, and the Wnt pathway are affected. In conclusion, CAFs play a significant role in CRC and impact the TME throughout development.

## 1. Introduction

Colorectal cancer (CRC) is a malignant neoplasm of the gut mucosa with localization either in the colon or rectum [1]. CRC is the third most common cancer (10%) and the second leading cause of cancer-related death (9.4%) worldwide [2]. The 5-year survival rate is approximately 90% for early-stage and 13% for late diagnosis [3]. CRC development and progression are closely associated with cancer-associated fibroblasts (CAFs). A high stromal content on the invasive front of CRCs has been shown to correlate strongly with an increased risk of CRC-related death [4]. The consensus molecular subtypes [5] showed that the predictive values are mainly attributed to genes expressed by stromal cells rather than tumour cells. The CMS4 subtype is characterized by high stromal infiltration and is associated with the worst survival outcomes. Patients with inflammatory bowel disease have an increased risk of developing CRC due to the increased inflammatory signs associated with chronic bowel inflammation [6].

The TME comprises approximately 60–90% of the total tumour mass, and CAFs form the most abundant fraction. CAFs and other elements of the TME can develop a reciprocal relationship to maximize tumour fitness. CAFs influence cancer cell proliferation, tumour immunity, angiogenesis, ECM remodeling, and metastatic dissemination [7]. Activated CAFs adopt a contractile and proliferative phenotype and produce various ECM proteins, stiffening the EMC by forming collagen, promoting tumour growth, and creating a positive feedback loop for CAF activation. This shapes the TME via processes such as paracrine and autocrine signal transduction, producing immunosuppressive, angiogenic, and inflammatory factors [6,8]. Specific markers are required to detect CAFs in tumours, including smooth muscle actin (α-SMA), fibroblast activation protein (FAP), fibroblast-specific protein 1 (FSP1), andplatelet-derived growth factor receptor-α/β (PDGFRα or PDGFRβ). Fibroblast activation protein α (FAPα), a type II transmembrane serine protease, is expressed on the cell surface of fibroblasts and is now used as a universal marker for CAFs. It involves various physiological processes, including tissue remodeling and tumour progression [8,9,10].

The exact biological role of CAFs in the formation of CRC-associated stroma, tumour-promoting inflammation, and TME in general has not yet been investigated in detail. This work aims to provide a deeper insight into these relationships and their potential clinical value, particularly by investigating the malignancy marker FAP. Additionally, this work investigates the differences in CRC-related tumourigenesis, among other things, regarding the different signalling pathways of CRC development and embryogenesis.

## 2. Materials and Methods

### 2.1. Patient Cohort and Study Design

This study investigated a cohort of 155 formalin-fixed and paraffin-embedded (FFPE) specimens from 2001 to 2021, provided by the Institute of Pathology of the University Hospital Essen (Essen, Germany) archives. Tumours, independent of localization (*n* = 65; coecum, ascending, transverse, descending, sigmoid, and rectum), polyps (*n* = 37; 26 low grade and 11 high grade), and chronic inflammation (*n* = 20), as well as healthy samples (*n* = 33), were included in the analysis. Additional covariates included the patient’s age (median age 65 years), sex, location, TNM status, inflammation status, microsatellite instability (MSI), and the immune expression of the markers CD3, p53, BCAT, and Ki67 (Supplementary Table S1). A mutation profiling of CRC patients was performed to identify and categorize mutation-driven subpopulations by next-generation sequencing using a customized, targeted, amplicon-based sequencing panel established for routine diagnostic purposes (Supplementary Table S2). Furthermore, we performed a digital gene expression analysis via the NanoString nCounter^®^ system, utilizing a panel covering genes linked to the signalling pathways of tumourigenesis (MAPK, PI3K/Akt, TGF-β, Wnt, p53). In addition, the immunohistochemical results for FAP scoring were evaluated.

The study complied with the principles of the Declaration of Helsinki and was approved by the local ethics committee of the Medical Faculty of the University of Duisburg-Essen (protocol number 20-9141-BO). As no clinical data were implemented for analysis, and due to the retrospective nature of the study, the need for written informed consent was waived by the ethics committee.

### 2.2. Isolation and Quantification of Nucleic Acids

DNA and RNA were purified from 10 µm thick FFPE sections using the Maxwell^®^ RSC DNA FFPE Kit or Maxwell^®^ RSC RNA FFPE Kit (AS1440, Promega, Madison, WI, USA), according to the manufacturer’s recommendations. RNA was eluted in 50 µL nuclease-free water and stored at −80 °C, and DNA was eluted in 50 µL nuclease-free water and stored at −20 °C.

The fluorometric quantification of DNA and RNA was performed using the Qubit™ 4 fluorometer. The Qubit™ RNA Broad Range Assay Kit (Q10210, Thermo Fisher Scientific, Waltham, MA, USA) was used for RNA, and the Qubit™ dsDNA BR Assay Kit was used for DNA. For each sample, 1 µL of the isolated nucleic acids was used.

### 2.3. Digital Gene Expression

NanoString nCounter^®^ technology was used for digital RNA expression pattern analysis. Samples were prepared following the manufacturer’s instructions (MAN-10056-3 January 2020, Code Set Hybridization Setup). The code set used for hybridization encompassed 76 target genes and 5 reference genes (*ACTB*, *B2M*, *GAPDH*, *RPL19*, and *RPLP0*). All probes synthesized and validated by NanoString (Seattle, WA, USA) are shown in Table 1. For data analysis, the sample cartridges from the prep station were transferred into the nCounter^®^ Digital Analyzer; the color codes for each target molecule were read using the high-sensitivity program and 555 FOV.

### 2.4. NanoString Data Processing

The raw data obtained from the nCounter^®^ Digital Analyzer were analyzed using the nSolver^®^Analysis software (v4.0) from NanoString^®^ for raw data generation and the statistical programming language and environment “R” (v4.1.0) (R Foundation, Vienna, Austria) for subsequent data processing and analysis. Considering the counts obtained for positive control probe sets, raw NanoString counts for each gene were subjected to a technical factorial normalization, carried out by subtracting the mean counts plus two-times standard deviation from the CodeSet’s inherent negative controls. Subsequently, a biological normalization was performed using the included RNA reference genes. Additionally, all counts with *p* > 0.05 after a one-sided Wilks *t*-test versus negative controls plus 2-fold standard deviations were interpreted as not expressed to overcome basal noise.

### 2.5. Next Generation Sequencing

The subsequent library prep was performed using QIAseq Targeted DNA Panel, QIAseq 96-Index I Set, GeneReadDNAseq Panel PCR Kit V3, and Agencourt^®^ AMPure^®^ XP Beads, as recommended by the manufacturer (QIAseq^®^ Targeted DNA Panel Handbook For the ultrasensitive, targeted, next-generation sequencing (NGS) of DNA for Illumina^®^ NGS systems, March 2021). The multiplex PCR and subsequent purification were performed using the GeneReadDNAseq Custom Panel V3, named the Comprehensive Cancer Panel (CCP3). DNA libraries’ quality and quantity were assessed using the D5000 Screen Tape assay on the Agilent 2200 TapeStation system (Santa Clara, CA, USA). The pooled library was sequenced on an Illumina MiSeq platform (2 × 150 bases paired-end run) using the MiSeq Reagent Kit v2 (Illumina, San Diego, CA, USA) and analyzed via the Cancer Research Workbench (CLC Bio, Qiagen, Hilden, Germany). A mean coverage of approximately 8000-fold was obtained for each sample. Within the CLC Cancer Research Workbench, de-multiplexed paired-end sequencing data were mapped to a human reference genome (hg19). Information on target regions from different databases (Cosmic, Clinvar, dbSNP, 1000 genome project, HapMap) was annotated. All variants found in Cosmic and/or Clinvar were listed as pathogenic. An allelic frequency with a minimum of 5% and a coverage of at least 100 mapped reads were used as selection parameters.

### 2.6. Immunohistochemistry

Hematoxylin-eosin (H&E) slide staining was conducted at a routine laboratory of the Institute of Pathology, University Hospital Essen, using the automated Stainer Ventana HE 600 (Roche, Basel, Switzerland). Immunohistochemical staining was performed according to our routine standard protocols) using an automated Stainer (Ventana Discovery XT). FAP was stained using the SP325 monoclonal antibody (Abcam, 48 min antigen retrieval in buffer with 60 min incubation time, dilution 1:100).

FAP expression was evaluated using the immune-reactive score (IRS). The overall percentage of the primary tumour with stroma FAP staining was evaluated semi-quantitatively (0, 1+, 2+, 3+), as well as the staining intensity (none, weak, moderate, strong) [11]. The IRS is composed of the number of positively stained cells (percentage point (PP)) and the staining intensity (IS). Both parameters, multiplied, result in the IRS (PP × SI) [12].

### 2.7. Statistical Evaluation

The statistics program R (v4.1.0) was used for the statistical and graphical analyses. The Shapiro–Wilks test was applied to monitor the Gaussian distribution of data. For ordinal variables, either the Wilcoxon–Mann–Whitney rank sum test for non-parametric variables or the two-tailed Student’s *t*-test for parametric variables was used. For ordinal variables with more than two groups, either the Kruskal–Wallis test for non-parametric variables or ANOVA for parametric variables was used to detect group differences. Double-dichotomous contingency tables were analyzed using Fisher’s exact test. To test the dependency of ranked parameters with more than two groups, the Pearson’s Chi-squared test was used. Correlations between metrics variables were tested by applying Spearman’s rank correlation test, as well as Pearson’s product–moment correlation testing for linearity.

A basic quality control of the data that were used was performed by mean-vs.-variances plotting to find outliers in the target or at the sample level. True differences were calculated by correlation matrices analysis. Pathway analysis is based on the KEGG database and was performed using the “pathview” package in R. Differences were specified by −log2 fold changes between the means (if parametric) or medians (if non-parametric) of the compared groups. Significant pathway associations were identified by gene set enrichment analysis using the WEB-based Gene Set Analysis Toolkit (WebGestalt) [13,14]. Differences between feature groups were estimated as log2 fold-changes. KEGG was also used as the database providing the functional categories for enrichment analysis. The minimum number of genes for a category was set as 5. Redundancy reduction was performed by affinity propagation. FDR was calculated using the Benjamini–Hochberg procedure. Each run was executed with 1000 permutations.

Finally, all associations were ranked according to the false discovery rate (*p* < 0.05). Due to the multiple statistical tests, the *p*-values were adjusted by using the false discovery rate (FDR). The level of statistical significance was defined as *p* ≤ 0.05 after adjustment.

## 3. Results

### 3.1. Distribution of FAP in Clinical Samples via Immunohistochemistry

**Figure 1:**
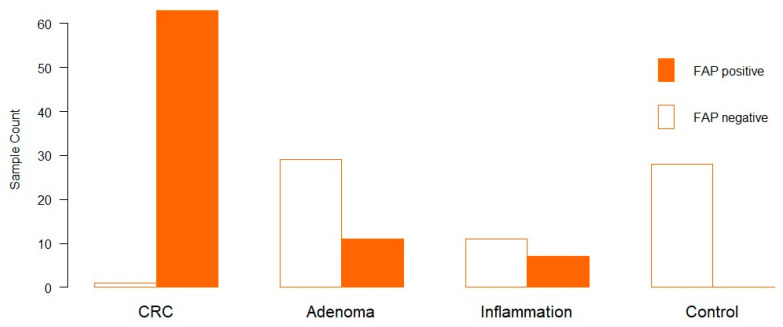
FAP expression in clinical samples (x-axis). The y-axis shows the number of samples. The Orange bar indicates FAP expression, and the white bar indicates no FAP expression.

We identified the expression of FAP in 81 of 150 samples. FAP expression was identified exclusively by cytoplasmic staining. In 98.4% of the CRC cases, FAP expression was observed. A total of 27.5% of adenoma patients showed positive FAP expression. Seven out of eighteen patients with inflammation expressed FAP (38.9%); however, there was no expression of FAP in the control group (Figure 1).

Regarding the percentage of FAP-positive fibroblasts, the overall mean value was 24.27%, and the overall median was 5% (Supplementary Figure S9). CRC showed a mean of 47.3% and a median of 45%. On the other hand, Adenomas showed a mean of 20.7% and a median of 0%, whereas there were 26 low-grade and 11 high-grade adenomas. Those positively stained adenomas were, with one exception, all high grade. This was followed by chronic bowel inflammation with a mean of 10.7% and a median of 0%. In line with this, CRC has the highest IRS, with 98.41%, followed by inflammation, with 38.89%. In adenomas, the intensity of FAP was about 37.93%.

### 3.2. Comparison of the Gene Expression Patterns of Tumours with Low and High Infiltration by FAP-Positive Fibroblasts

**Figure 2:**
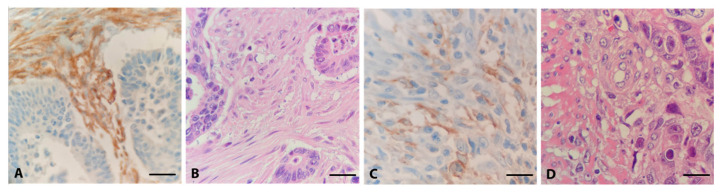
Different expression patterns of FAP in CRC. Representative examples of high (**A**) and low (**C**) immunohistochemical expression of FAP, with corresponding H.E. slides (**B**,**D**), 40×.

The rationale for comparing CRC with low- and high-infiltrating FAP-positive fibroblasts was to identify the signalling pathways that CAFs may influence. Figure 2 shows representative FAP staining analyzed by immunoreactive score (IRS) to evaluate the high and low scores of FAP expression.

A total of 13 out of the 74 examined genes showed a significant association with the occurrence of FAP-positive fibroblasts in CRC cases. Eight of the thirteen studied genes were highly expressed (*FAP*, *FN1*, *TGFBR1*, *WNT2*, *CDK4*, *TGFB1*, *CDK1*, and *WNT3*), while five of the genes were downregulated (*PIK3R1*, *FZD5*, *SOS2*, *EGFR*, and *PTEN*) in samples infiltrated by CAFs (Supplementary Table S3). The significant targets associated with the amount of activated fibroblasts (in %) include directly associated targets such as *FAP*, *FN1*, *TGFB1* and *WNT2*. Indirectly associated targets are *PIK3R1*, *SMAD4*, *ARAF*, *SOS2* and *BECN1* (Supplementary Table S4).

The calculated IRS score for FAP expression was compared with all target genes. Significantly upregulated genes were *FAP*, *FN1*, *TGFB1*, and *Wnt2*, whereas *ARAF*, *PIK3R1*, *SMAD4*, *BECN1*, and *EGFR* were downregulated (Supplementary Table S5).

A gene set enrichment analysis (GSEA) of FAP expression was performed to identify the biological background mechanisms, pathways, and biological functions behind the different expression patterns associated with FAP-positive fibroblasts. GSEA showed a prominent enrichment of pathways related to *PI3K* (Supplementary Figure S3) and MAPK signalling. Still, immune response pathways such as NK-cell-mediated cytotoxicity are activated via CAFs. Details of the GSEA, including the normalized enrichment value, the *p*-value of enrichment, the precise targets contained in the gene sets, and those that are differentially regulated, can be found in Supplementary Tables S7–S9 and Figures S5–S7.

### 3.3. Adenoma vs. CRC

Digital gene expression analysis was focused on CRC and adenomas to enlighten the association between CRC and its precursor lesions. Sixteen out of the seventy-four analyzed genes showed a significant association with the occurrence of adenomas or CRC. Of these, nine were highly expressed in CRC (*FN1*, *FLT4*, *VEGFA*, *Wnt3*, *TGFB1*, *PDGFB*, *CCND1*, *TGFBR1*, *FAP*), while seven were downregulated (*PTEN*, *SMAD4*, *EGFR*, *ATG14*, *FZD5*, *NRAS*, *BRAF*) (Supplementary Table S6; GSEA Figure S8 and Table S10).

## 4. Discussion

### 4.1. Active Fibroblasts Are Abundant in Colorectal Cancer

We could show that almost all (98.4%) of our investigated samples of invasive CRC exhibited FAP-expressing fibroblasts. Inflammatory tissue was displayed in 38.9%, and the adenomas showed 27.5% FAP expression, whereas controls were completely devoid of FAP expression. The FAP expression level within carcinoma is in line with previous studies, which showed that FAP is expressed in more than 90% of cases [11,15,16]. We proved that CAFs play an important role in CRC and that almost all our tumour samples contained CAFs. In 1990, one study detected FAP in all CRC samples, and one out of seven adenomas showed high FAP expression [17]. Other studies showed a 24% FAP expression in adenomas or no expression at all [18,19]. This indicates that the recruitment and activation of fibroblasts are not part of the initial tumourigenesis, but more likely play a role at a later stage when tumour cells become progressively malignant with invasion and metastatic spread. We hypothesize that the recruitment of CAFs might be a late and possible final step into invasive malignancy, and that the positive adenomas at the respective sites have already started the transformation process. This conclusion is also strongly supported by the previously mentioned observation, in which FAP-positive fibroblasts were found only in the transition zone of high-grade adenomas. This hypothesis is also taken up in numerous publications describing the activation of the stromal cells, like fibroblasts, as already occurring in the pre-invasive stages of tumourigenesis. All these facts combined indicate that FAP can be a powerful diagnostic tool for estimating borderline invasiveness.

In addition, it has been reported that the tumour stroma is already prepared for the subsequent stages of tumour development, providing a “seed fertilizing soil”, according to Kuzet and Gaggioli [7]. The close reciprocal relationship between tumour cells and CAFs creates a tight interaction mechanism. Tumour cells promote the formation of CAFs and maintain their activated state. The constant activation of CAFs can support tumour cell growth, migration, and invasion by reprogramming and remodeling the tumour stroma. This ultimately leads to tumour progression, metastasis, and resistance to therapy [20].

In addition to malignant lesions, FAP-positive cells could also be observed in active inflammatory conditions. During wound healing, fibroblasts are activated and become myofibroblasts, which attempt to restore tissue architecture and homeostatic states prior to injury [7].

### 4.2. CAFs Have a Major Influence on the Cell Cycle Signalling Pathway

**Figure 3:**
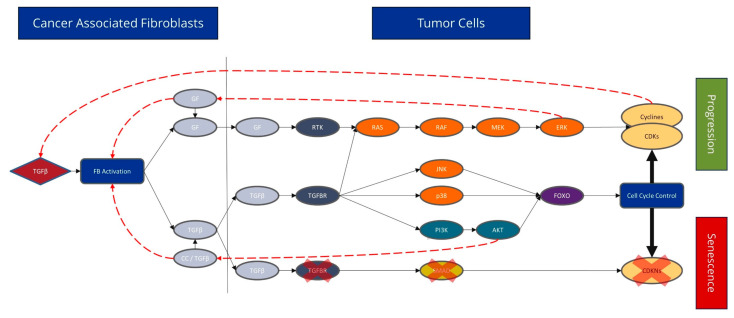
The expression of different signalling pathways involved in TGF-β (Black solid arrows). Tumour cells produce TGF-β, which acts on the fibroblasts via the TGFB1 receptor, and the actual canonical pathway is completely switched off in fibroblasts. TGF-β is responsible for apoptosis and G1 arrest of cells; fibroblasts become persistent after switching the pathway off (marked by the red X). TGF-β expression may also lead to non-canonical TGF-β signal transduction via the p38 MAPK. As a result, fibroblasts secrete various growth factors, which trigger mitogenic signalling in the tumour (red dashed arrow). Subsequently, cell cycle activation causes cells to move from the G1 phase directly to the S phase. Apoptosis and cell cycle arrest are particularly affected by CAFs.

In general, we found substantial differences in the dependence of CAFs on the biology of CRC. In summary (Figure 3), we hypothesize that emerging tumour cells produce releasing transforming growth factor β (TGF-β), which acts on the fibroblasts via the TGFB1 receptor; this could completely switch off the actual canonical pathway in the fibroblasts. TGF-β is responsible for apoptosis and the G1 arrest of cells, so fibroblasts become persistent after switching off the pathway. Fibroblasts are present within tissue in a dormant state and are activated by TGF-β in wounds or during inflammation. During wound healing, activated myofibroblasts contract, resulting in the closure of the wound edges. In chronic wounds, active fibroblasts can produce TGF-β themselves. Furthermore, myofibroblasts stimulate angiogenesis and thus ensure the supply of newly formed tissue. After wound closure, the myofibroblasts disappear from the newly formed tissue by undergoing apoptosis or return to a quiescent state of healing [7,21,22]. The expression of *TGF-β* can lead to non-canonical TGF-β signalling through p38 MAPK. This causes fibroblasts to release various growth factors that trigger mitogenic signals in the tumour. As a result, the cell cycle is activated, causing cells to move directly from the G1 phase to the S phase. TGF-β acts as a cell cycle inhibitor, which slows or stops cell growth, promoting cell survival signalling [23] (Supplementary Figure S1).

In addition, cyclin-dependent kinases 4 and 1 (CDK), together with their ligand, cyclin D1 (CCND1), play an essential role in cell cycle regulation (Supplementary Figure S2). CDKs regulate the transition to the different phases of the cell cycle [24,25]. Myc organizes various cellular tumour functions, including the cell cycle and cell proliferation. C-Myc is essential in CRC and is significantly upregulated in 70% of all CRC cases [26]. Cell cycle proliferation is further enhanced by active Wnt signalling, whereby the actively enslaved fibroblasts produce Wnt, and the tumour cells activate the membrane receptor Frizzled. In a majority of CRC cases (90%), mutations in Wnt signalling are predominant in early development [27].

According to the data outlined above, the life cycle of fibroblasts can be considered in the context of tumour activation [7]. In the healthy colon, fibroblasts usually rest, with reduced transcriptomic activity. They have a significant role in tissue integrity and the maintenance of homeostasis. The origin and development of desmoplastic transformation and recruitment of myofibroblasts, particularly their exclusive presence in malignant lesions and absence in (still) benign precursor stages, remains unresolved. Although this descriptive study, relying solely on clinical samples, without functional assays, does not provide a definitive explanation for the chicken-or-egg principle, there is a suspicion that a fundamental mechanism involving the constitutive activation of either *KRAS* or *BRAF* mutation plays a crucial role in inducing the classical MAPK signalling pathway. The subsequent secretion of primary growth factors and subsequent secretion of TGF-β would probably trigger and sustain the vicious cycle, potentially addressing the above questions. This process is set in motion by the subsequent cascade that was outlined, thereby instigating the conversion of CAFs.

Their activation is the primary trigger during the initial life cycle phase, involving fibroblasts and other progenitor cells. Subsequently, the fibroblast undergoes a transformative process, leading to cancer-associated fibroblast (CAF) formation. The disruption of the basement membrane characterizes this desmoplastic reaction. A reciprocal dialogue with the microenvironment is possible and leads to the remodeling of the tissue structure. The relationship between CAFs and tumour-promoting inflammation is also important, as CAFs can interact with immune cells to promote a pro-inflammatory environment. This occurs by releasing cytokines and growth factors that can stimulate inflammatory responses and thereby support tumour growth, which is particularly prevalent in the desmoplastic response [28,29].

In the case of extensive stroma restructuring, CAFs mature and undergo cell senescence. This reduces the proliferation and differentiation capacity of senescent CAFs while the secretion capacity of CAF precursor cells increases [7,30]. The signalling pathways discussed above can be described in the individual stages to underline the life cycle. CDK1/4 shows the above-described effect only in the late stage, when stroma remodeling is completed and CAFs are senescent [31]. The TGF-β receptors and the canonical TGF-β signalling pathway seem to already be present from the beginning of the process, which could underline its importance as an initiator [32]. TGF-β exhibits two distinct activation patterns associated with CAFs. The first activation pattern can be observed at FAP intensity 0–2, related to fibroblast activation. In the further course, TGF-β shows even higher activity than in the first step (FAP intensity 3) after the completion of stroma remodeling. This increased expression of TGF-β could be interpreted by the fact that the recruited fibroblasts mature during tumourigenesis and could then provide TGF-β and other secreting factors on their own. *TGF-β* is a tumour suppressor in the early stages of tumourigenesis, inhibits cell proliferation, and induces apoptosis. However, it serves as a tumour promoter in the late stages and stimulates invasion, angiogenesis, and immunosuppression [32]. *Wnt3* is directly present at the initiation phase, with *Wnt2* appearing at the end to support the proliferation of cells in the tumour. *Wnt2* supports invasion and metastasis in CRC through the autocrine activation of the canonical Wnt pathway in CAFs [33] (Supplementary Figure S4).

### 4.3. Different Expression Patterns of CRC and Adenomas

**Figure 4:**
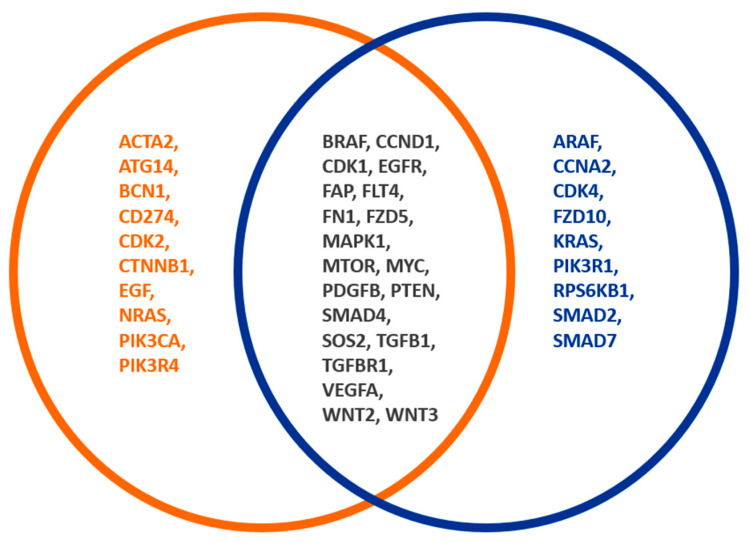
Different FAP expression patterns of CRC and adenomas. The orange circle shows genes of CRC and adenomas, whereby the blue circle shows the genes of FAP-positive fibroblasts. The overlapping gene expression between both groups is indicated by a black font. A total of 16 out of 74 analyzed genes showed a significant association with the occurrence of adenomas or CRC. In comparing CRC and adenomas, the CDK (cell cycle), TGF-ß, and Wnt signalling pathways are activated in CRC by CAFs.

Different expression patterns could be found in adenoma and CRC, but the processes of active fibroblasts could not be assessed. It would be interesting to address this in future research. In comparing CRC and adenomas, the CDK (cell cycle), TGF-ß, and Wnt signalling pathways are assumed to be activated in CRC by CAFs. In addition, there were other changes that we could not relate to the fibroblasts, as they did not show any difference in the comparison of tumours with and without FAP-positive fibroblasts (Figure 4).

This mainly concerns the MAP kinase signalling pathway starting with an increased expression of *SOS1/2*, which is higher in adenoma than in CRC. The expression levels of the RAS genes, particularly *NRAS*, as well as *BRAF*, increased. Several papers have shown that particularly serrated polyps usually undergo a genetic alteration to activate the MAPK pathway. Most commonly, this occurs due to *KRAS* or *BRAF* mutations [34,35,36], thereby initiating RAS-RAF-MEK-ERK and RAS-PI3K-PDK1-AKT signalling pathways, both of which are essential for cell survival and proliferation [37]. Interestingly, MYC and ERK showed no substantial difference, with ERK showing a slight trend towards higher expression (*p*-value 0.03, FDR adj. P 0.09). We explain this slightly increased expression of the MAP kinase by the constitutive activation of this signalling pathway, primarily by mutations in *RAS* or *RAF* genes. The carcinoma is, therefore, not dependent on the increased expression. However, it is conceivable that adenomas have a slight growth advantage over normal tissue due to the overexpression of essential genes. The interaction between fibroblasts and adenomas has not yet been comprehensively explored at the molecular level. Most conclusions are based exclusively on microscopic and immunohistochemical analyses [38].

### 4.4. The Detrimental Impact of CAFs on Clinical Course, Survival Rate, and Treatment Response

However, our study focused on the biological consequences of TMEs, particularly CAFs, rather than tumours’ clinical behavior; many studies have shown that CAFs play a significant role in proliferation, invasion, tumour progression, and angiogenesis [38,39,40,41]. This occurs, among other things, through direct cell–cell contact or indirect paracrine signalling between CAFs and cancer cells [22]. CAFs also become essential for growth factors, cytokines, chemokines, and exosomes [30]. In many studies, a high expression of FAP is associated with poor and aggressive progression. The association is controversial in rectal cancer and patients with high CIMP and FAP expression at the tumour front. Furthermore, there was also a high potential for developing metastases or recurrence [11,16]. The maturity of CAFs also plays a role in survival, as immature CAFs often tend to infiltrate tumours, and mature CAFs promote lymphatic invasion and show an expanding growth pattern [41,42].

CAFs can mediate therapy resistance by secreting soluble factors. They restructure the ECM to prevent macromolecules from penetrating rigid collagen fibers or mediate drug resistance through cell adhesion. Furthermore, increased interstitial fluid pressure in the stroma causes blood vessels to collapse, leading to hypoxia in the tumour. Hypoxia reduces the effect of oxygen- or radical-dependent therapeutic approaches. A promising therapeutic approach is FAP-directed therapy, which is present in up to >90% of CRC. Therapeutic approaches target FAP by inhibiting enzymatic function or introducing antibodies and immunotherapies into the tumour microenvironment [43,44]. Zafari et al. sought an overview of the TME in CRC, focusing on CAFs with potential therapeutic approaches [45]. Nevertheless, even here, it only emerged that CAFs play an essential role in tumour development, and there are no precise general therapies to counteract this, only various approaches. Combination therapies have shown a higher probability of success, particularly in vitro, although their efficacy in vivo still needs to be confirmed [46]. Of particular interest would be active substances that are also involved in the signalling pathways in the cell cycle, differentiation, and apoptosis, as shown in this paper.

For instance, a combination therapy comprising TGF-β inhibitors and anti-PD-L1 immunotherapy has proven effective. This is because TGF-β signalling in stromal cells decreases, which results in increased T-cell infiltration and improved antitumour immunity [47]. Cyclin-dependent kinases (CDKs) are a crucial therapeutic target in treating colorectal cancer (CRC) as they induce apoptosis. Flavopiridol can inhibit the expression of death receptor 5 (DR5) through p73, a member of the p53 family, and its efficacy is enhanced when used in combination with other chemotherapeutic agents such as gemcitabine or γ-radiation [46,48]. The inhibition of MEK in the MAPK signalling pathway also shows that triple therapy is more effective than monotherapy, as it combines the inhibition of BRAF and MEK with dabrafenib and trametinib, providing a significantly improved response and improved survival rates [49,50]. The current discourse indicates that there needs to be more research on CAFs, despite their exhibiting significant heterogeneity. Consequently, there is an urgent need for targeted therapy to address this issue [38].

### 4.5. Limitations of the Study

We successfully conducted a study about the effects of CAFs on the biology of CRC, but this only provided a rough overview and snapshot of its complex and heterogeneous nature. Due to the diversity of mutations and signalling pathways, different biology on the left and right sides, and various biological tumour entities in CRC, it is challenging to gain a clear understanding, as mentioned in Section 4.4. Moreover, the fluid transition of potentially premalignant lesion may affect the comparison of polyps and CRC samples. The direct effect of FAP, either positivity or generally, on the appearance of myofibroblasts on this progression has to be discussed, as well as the chicken–egg problem. While RNA bulk analysis can inform us about gene expression patterns, a clearer understanding is needed of the cellular and spatial distribution of these expressions. Despite relying on prior knowledge and attempting to create reasonable hypotheses, the study is limited in scope and only offers a snapshot of the more significant molecular dynamics. Such limitations include the use of FFPE samples and a small gene panel of 76 genes. These factors emphasize the need for further research to expand and refine our understanding of the complex dynamics within the studied parameters.

## 5. Conclusions

In this study, CAFs were shown to significantly impact the biology of CRC. An infiltration with FAP-positive fibroblasts was found in almost 100% of CRC, reflecting the importance of CAFs in CRC. They seem to play a significant role in tumour development and progression. CAFs secrete various signalling substances that have a paracrine action on the surrounding cancer cells, stromal cells, or autocrine. Specific gene expression patterns in the TGF-β signalling pathway could underline the importance of CAFs as key players in CRC biology. In addition, CAFs appear to affect cell cycle, progression, and control.

We present a detailed hypothesis of CAF activation due to tumour cells, a process of CAF maturation, and their regulatory functions on tumour progression and maintenance. Beginning with the TGFB-driven activation of non-canonical signalling via p38-MAPK and the MEK/ERK axis, it is hypothesized that CAFs may contribute to malignant features such as invasiveness and the G1/S phase transition. Regardless, a lot is still unknown about the regulatory mechanisms of CAF infancy and persistence. In addition, the biological background mechanisms of CRC are not fully understood. The interplay of CRC and CAFs harbors much potential for further studies concerning therapeutic approaches and the role of CAFs within the TME.

## Figures and Tables

**Table 1:** Gene list sorted by signal pathways included in the Codeset-designed panel for the NanoString nCounter.

Cell Cycle	PI3K Signalling Pathway	MAPK Pathway	WNT Signalling Pathway	Growth Factors	TGF-β	Fibroblast Markers
*CDK1*	*PIK3C3*	*MAP2K1*	*CTNNB1*	*EGFR*	*TGFB1*	*ACTA2*
*CDK2*	*ATG14*	*KRAS*	*Wnt1*	*TGFA*	*TGFBR1*	*FAP*
*CDK4*	*PIK3R5*	*MAPK1*	*Wnt2*	*EGFR*	*TGFBR2*	*FN1*
*CDK6*	*PIK3R4*	*BRAF*	*CD47*	*HGF*	*CD44*	
*MDM2*	*PIK3R1*	*CHRM3*	*CD274*	*FGF1*	*SMAD4*	
*TP53*	*PIK3CA*	*HRAS*	*ABCB1*	*VEGFA*	*SMAD2*	
*WEE1*	*RPS6KB1*	*NRAS*	*Wnt3*	*FLT1*	*SMAD7*	
*Myt1*	*BECN1*	*ARAF*	*FZD10*	*FLT4*	*MYC*	
*CDKN2A*	*AKT1*	*SOS1*	*FZD2*	*KDR*		
*CDKN1B*	*MTOR*	*SOS2*	*FZD5*	*PDGFB*		
*CDKN1A*	*PTEN*	*NF1*		*IGF1*		
*CCND1*	*RICTOR*	*MAP2K2*		*VEGFC*		
*CCNA2*	*RPTOR*	*RAF1*		*MET*		
*CCNB3*						
*CCNE1*						

## Data Availability

The datasets used and/or analyzed during the current study are available from the corresponding author on reasonable request. The data is not publicly accessible for data protection reasons.

## Supplementary Materials

The following supporting information can be downloaded at: https://www.mdpi.com/article/10.3390/genes15020209/s1, Table S1: Clinicopathological data of the cohort. Table S2: Genes included in the CCP3 panel for next-generation sequencing. Table S3: Differences in gene expression between tumors with and without infiltration of FAP positive CAFs. Table S4: Correlation analysis of gene expression compared to FAP positivity (in %). Table S5: Correlation analysis of gene expression and IRS score in FAP-positive samples. Table S6: Differential gene expression patterns between CRC and their precursor lesions. Table S7: Gene set enrichment analysis of differentially expressed genes based on FAP positivity. Table S8: Gene set enrichment analysis in FAP-positive fibroblast samples differentiated by low and high FAP expression (%). Table S9: Gene set enrichment in dependence of high and low FAP staining intensity. Table S10: Gene set enrichment analysis differentiated by adenoma and CRC samples in conjunction with CAFs. Figure S1: Analysis of the TGF-B signalling pathway. Figure S2: Analysis of the p53 signalling pathway. Figure S3: Analysis of the pi3k-akt signalling pathway. Figure S4: Analysis of the WNT signalling pathway. Figure S5: Gene set enrichment analysis of differentially expressed genes by positive Fibroblast. Figure S6: Gene set enrichment analysis for positive Fibroblast in high and low FAP percent expression. Figure S7: Gene set enrichment in dependence of high and low-intensity FAP. Figure S8: GSEA in dependence of adenoma and CRC samples. Figure S9: Correlation of FAP positivity in percent with all types.
