# Supplementary material for: The Impact of Cancer-Associated Fibroblasts on the Biology and Progression of Colorectal Carcinomas
Source: Genes (Basel). 2024 Feb 6;15(2):209. doi: 10.3390/genes15020209 (PMC10888097; doi:10.3390/genes15020209)
Supplement: Supplementary file 1 [file genes-15-00209-s001.zip › Supplement-Figure.pdf]

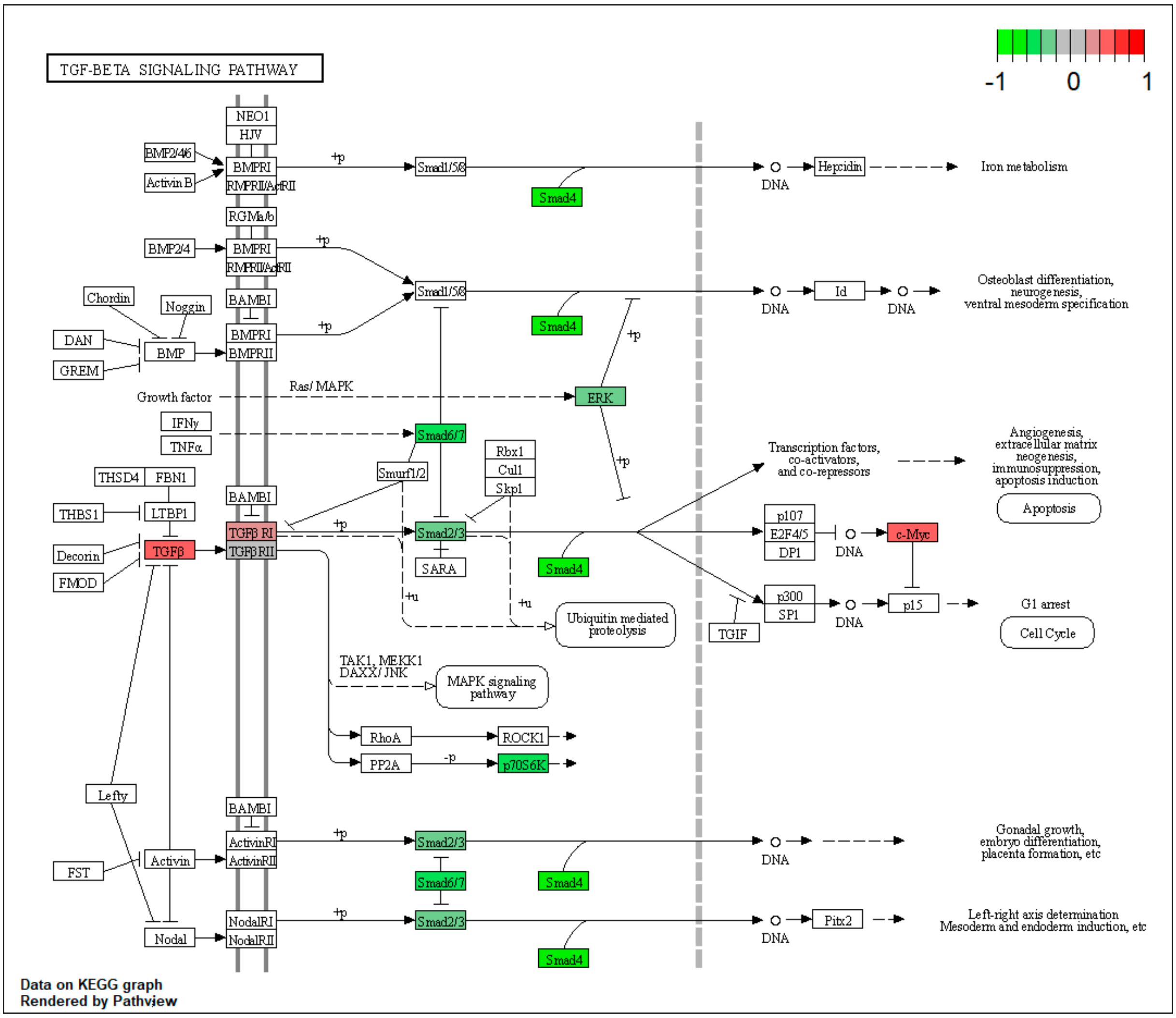

Figure S1: Analysis of the TGF-B signalling pathway. Tumor presenting CAF infiltration in overexpress target genes marked red and reduced expression of target genes marked in green. Apoptosis and cell cycle arrest are particularly affected by CAFs.

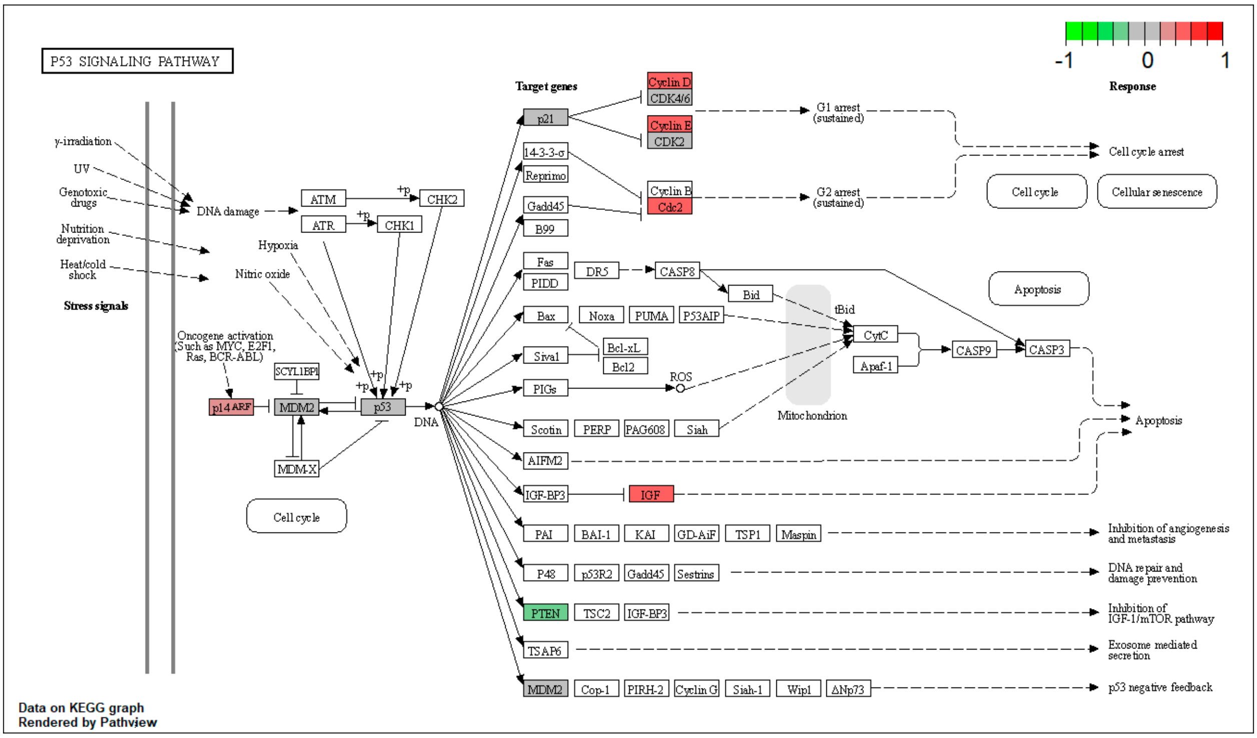

Figure S2: Analysis of the p53 signalling pathway. Tumor presenting CAF infiltration in overexpress target genes marked red and show reduced expression of target genes marked in green.

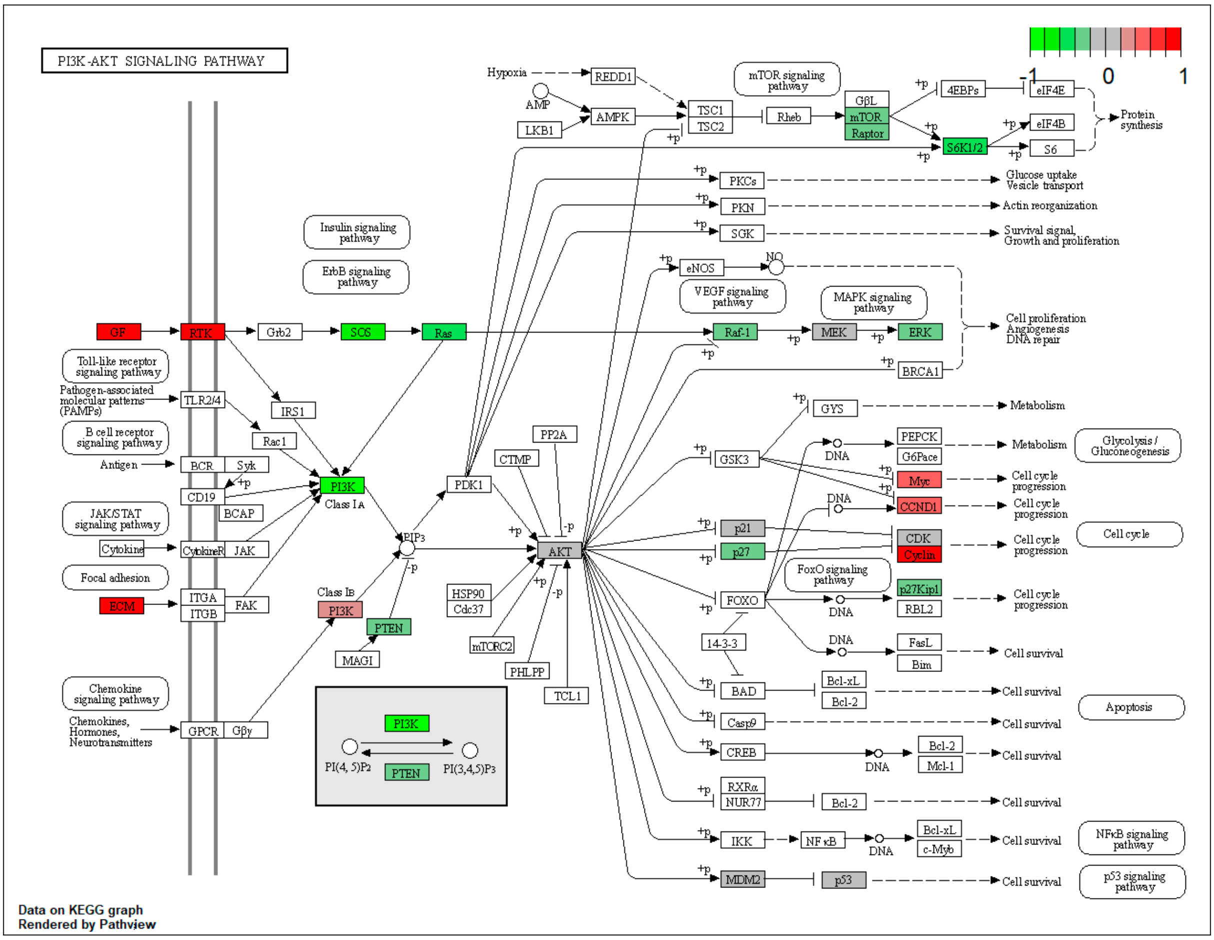

Figure S3: Analysis of the pi3k-akt signalling pathway. Tumor presenting CAF infiltration overexpress target genes marked in red and show reduced expression of target genes marked in green.

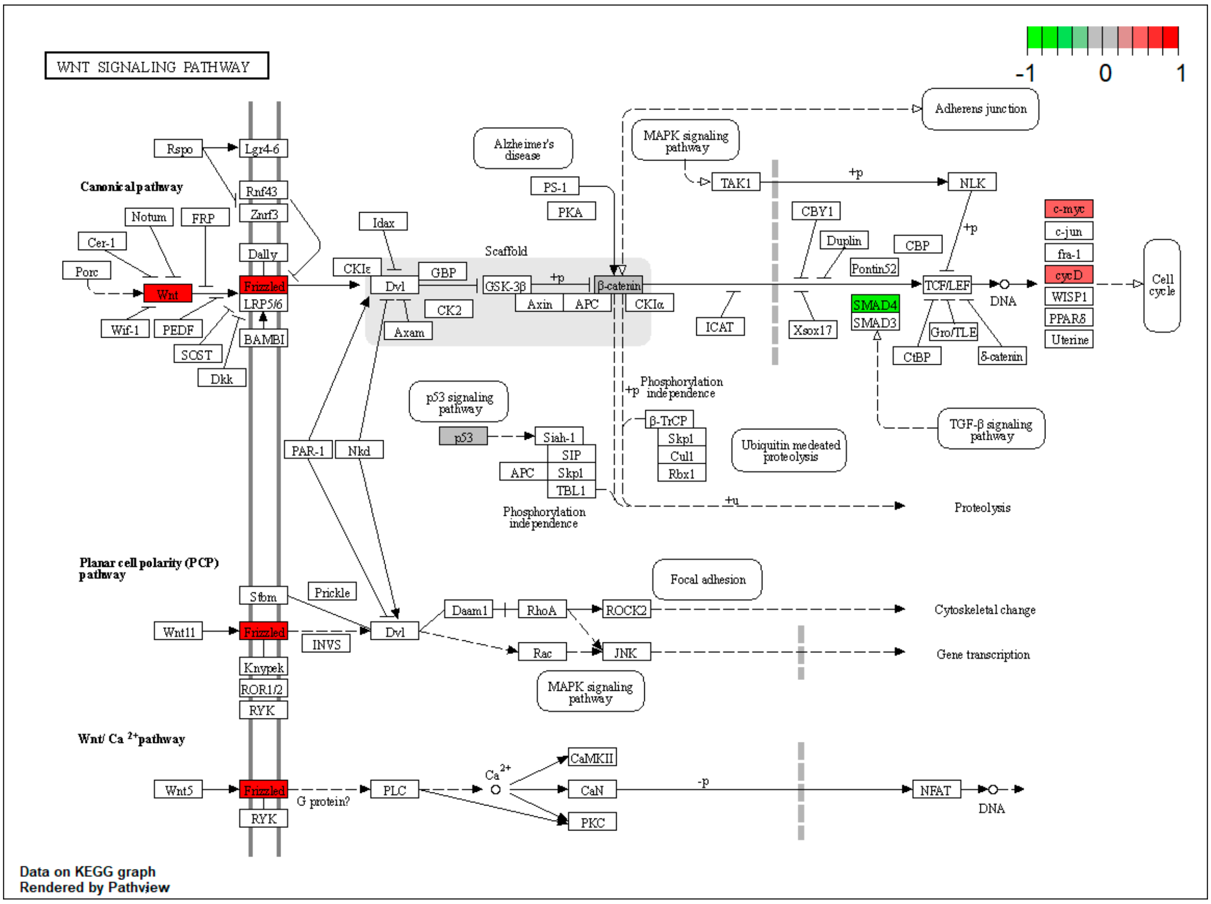

Figure S4: Analysis of the WNT signalling pathway. Tumor presenting CAF infiltration overexpress target genes marked in red and show reduced expression of target genes marked in green.

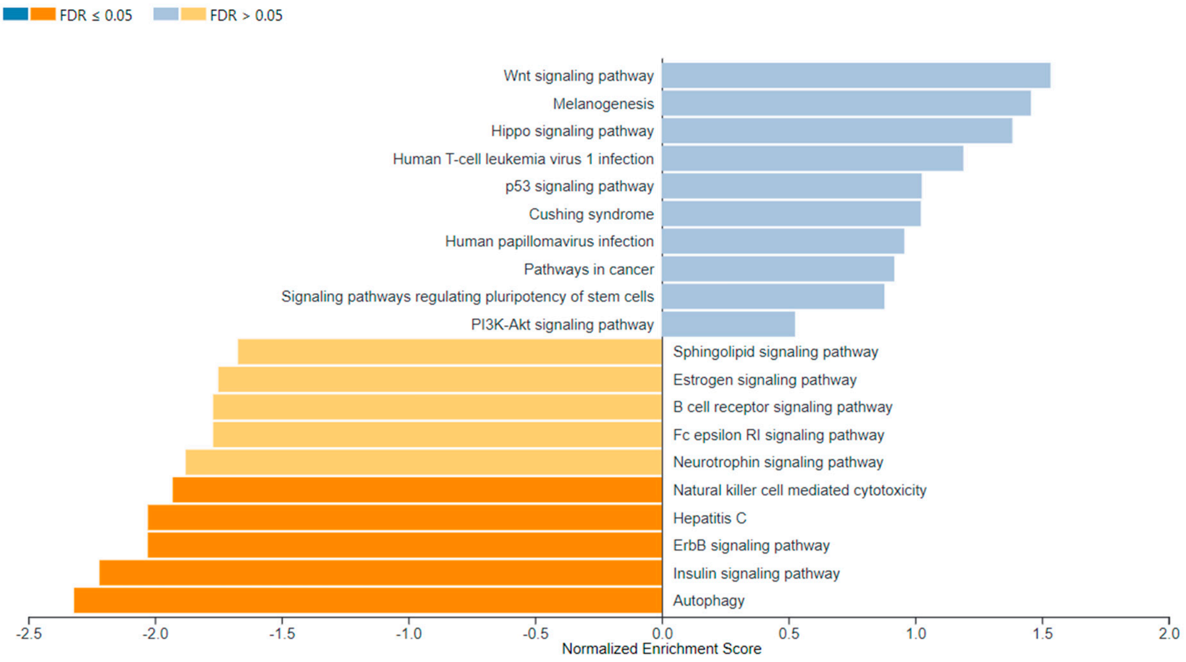

Figure S5: Gene set enrichment analysis of differentially expressed genes by positive Fibroblast presenting an overview of low gene sets enriched in Fibroblast (right side, blue bars) and strongly enriched gene sets (left side, yellow bars). The orange bars (left side) indicate significantly decreased enrichment (FDR-corrected p-Value < 0,05).

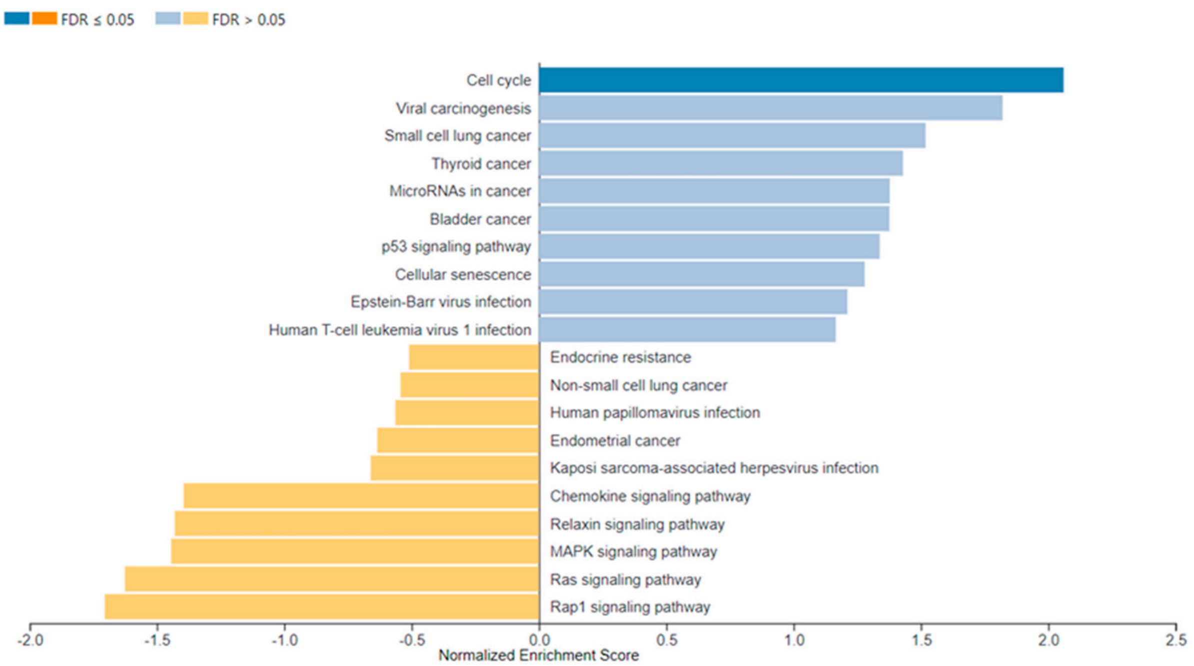

Figure S6: Gene set enrichment analysis for positive Fibroblast in high and low FAP percent expression. The differentially expressed genes by low percent of FAP enriched gene sets (right side, blue bars) and high percent of FAP enriched gene sets (left side, yellow bars). The dark blue bar (right side) indicates significantly increased enrichment (FDR-corrected p-Value < 0,05).

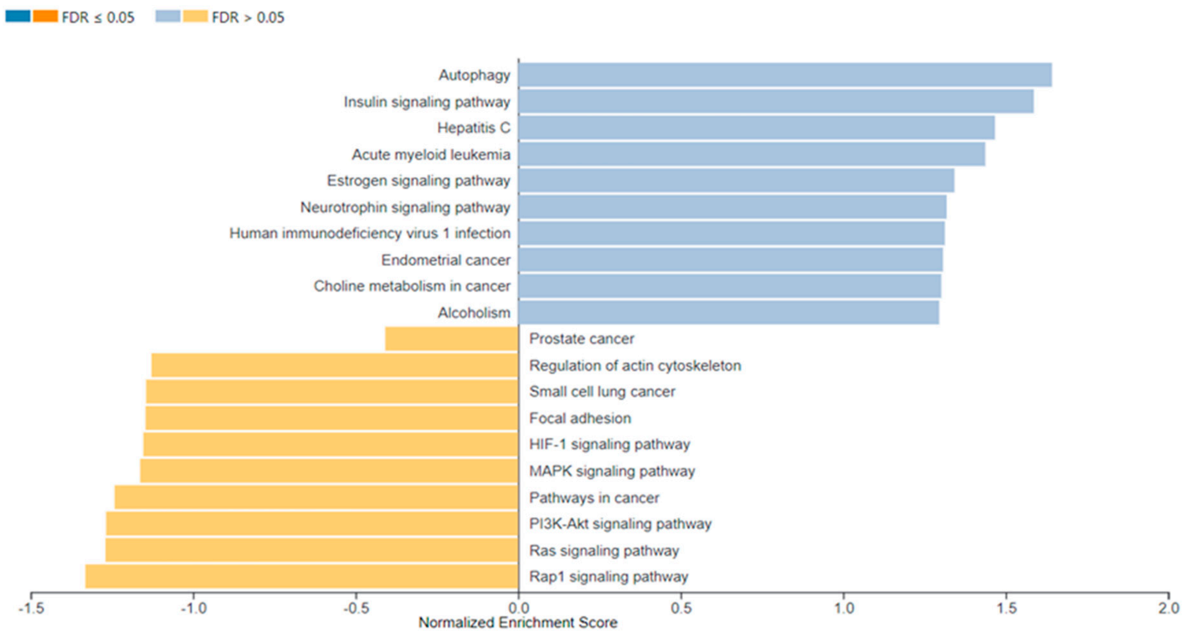

Figure S7: Gene set enrichment in dependence of high and low-intensity FAP. The right side (blue bars) gives an overview of low intensity, and the left side (yellow bars) represents the high intensity of FAP expression.

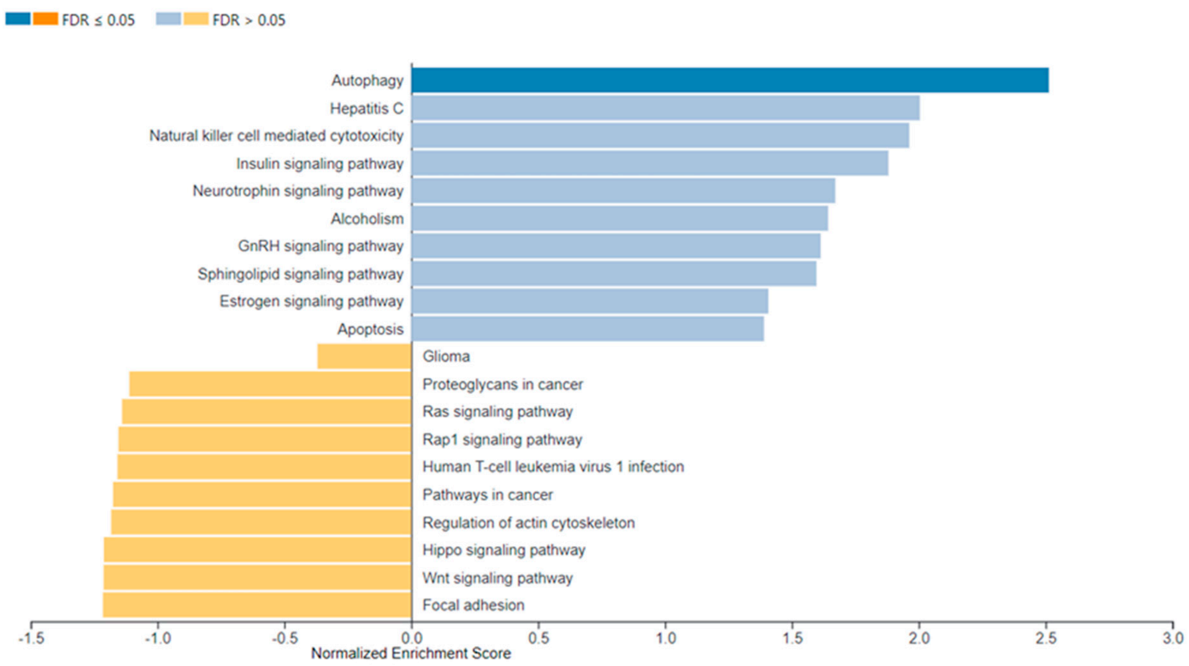

Figure S8: GSEA in dependence of adenoma and CRC samples. On the left (yellow bars), genes are strongly enriched in CRC. On the right side (blue bars) genes are strongly enriched in adenomas. Highly significant associations with an FDR-corrected p-Value of< 0.05 are highlighted by dark shaded colours.

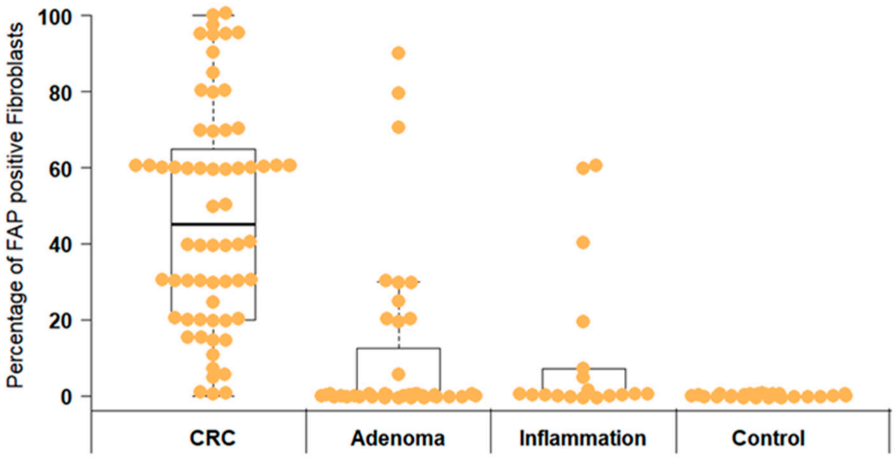

Figure S9: Correlation of FAP positivity in percent with all types. The x axis displays the different types. The y axis displays measured counts of FAP positivity in percent.
