# Supplementary material for: The Impact of Cancer-Associated Fibroblasts on the Biology and Progression of Colorectal Carcinomas
Source: Genes (Basel). 2024 Feb 6;15(2):209. doi: 10.3390/genes15020209 (PMC10888097; doi:10.3390/genes15020209)
Supplement: Supplementary file 1 [file genes-15-00209-s001.zip › Supplement-Table.pdf]

Table S1: Clinicopathological data of the cohort.

**age**	Min.	15	**TNM-Status**		**Mutation Status**	(K/H/N) - RAS	9
	Median	65	**T**			PIK3CA	7
	Max.	94	T1	9		TP53	19
**sex**	Male	76	T2	9		HER2	1
	Female	79	T3	38		BRAF	16
**type**	CRC	65	T4	7		TGFB	3
	Adenom	37	**N**		**MSI**	stable	12
	Inflammation	20	N0	38		high	10
	Control	33	N1	14			
	Total	155	N2	9			
**Lokalisation**	descendens	32	**M**				
	ascendens	44	M0	22			
	sigma	40	M1	12			
	transversum	13	**grade**				
	Rektum	21	G1	2			
	coecum	3	G2	52			
	Flexura Hepatica	2	G3	15			

Table S2: Genes included in the CCP3 panel for next-generation sequencing

**GENE**	**EXON**
BRAF	11, 15
EGFR	18-21
ERBB2	2, 3, 12, 17, 20, 26
FGFR1	3, 7, 13, 17
FGFR3	7, 9
HRAS	2-4
IDH1	4
KIT	9-11, 13, 17, 18
KRAS	2-4
MET	3, 8, 11, 14, 19
NRAS	2-4
PDGFRA	12, 14, 18
PIK3CA	3, 5, 10, 16, 21
RET	7, 10, 11, 13-16
STK11	1-9
TP53	2-11

Table S3: Differences in gene expression between tumors with and without infiltration of FAP positive CAFs. Significant correlations are indicated by an FDR-corrected p-Value of < 0.05. The confidence interval (CI) indicates whether the genes are highly expressed in tumors (negative) or whether they are down-regulated (positive).

**Gene**	**p-Value**	**FDR adjusted p-Values**	**lower CI**	**higher CI**
FN1	0.0008	0.018	-10591	-1820
FAP	0.0002	0.007	-556	-168
TGFB1	0.0059	0.04	-901	-164
TGFBR1	0.0028	0.035	-500.9	-130.6
CDK1	0.006	0.04	-418	-93
CDK4	0.0047	0.04	-423.6	-92.5
WNT2	0.0036	0.038	-185	-46
WNT3	0.0086	0.049	-28	-4
EGFR	0.0055	0.04	36	165
FZD5	0.001	0.018	97	316.5
SOS2	0.0016	0.024	120.8	403.6
PIK3R1	0.0001	0.006	377	800
PTEN	0.0075	0.046	152.1	824

Table S4: Correlation analysis of gene expression compared to FAP positivity (in %). Only correlations considered significant are displayed. One measure of evidence is an FDR-corrected p-Value of < 0.05. The Spearman's rank correlation coefficient (Rho) describes whether the genes are directly or in-directly associated with one another.

**Gene**	**p-Value (Spearman)**	**FDR adjusted p values (Spearman)**	**Rho**
PIK3R1	3.00E-04	0.00814	-0.39
SMAD4	0.00086	0.01332	-0.37
ARAF	9.00E-04	0.01332	-0.36
BECN1	0.0057	0.046866667	-0.31
SOS2	0.0051	0.046866667	-0.31
WNT2	0.0047	0.046866667	0.31
TGFB1	0.002	0.024666667	0.34
FN1	0.00033	0.00814	0.39
FAP	6.40E-06	0.0004736	0.48

Table S5: Correlation analysis of gene expression and IRS score in FAP-positive samples. Only correlations considered significant are displayed. One measure of evidence is an FDR-corrected p-Value of < 0.05. The Spearman's rank correlation coefficient (Rho) describes whether the genes are directly or indirectly associated with one another.

**Gene**	**p-Value (Spearman)**	**FDR adjusted p-values (Spearman)**	**Rho**
Intensity FAP (0 - 3) *PIK3R1*	0.00051	0.011963333	-0.38
Intensity FAP (0 - 3) *ARAF*	0.00074	0.011963333	-0.37
Intensity FAP (0 - 3) *SMAD4*	0.0018	0.019028571	-0.35
Intensity FAP (0 - 3) *BECN1*	0.0045	0.041625	-0.32
Intensity FAP (0 - 3) *EGFR*	0.0052	0.042755556	-0.31
Intensity FAP (0 - 3) *TGFB1*	0.00097	0.011963333	0.36
Intensity FAP (0 - 3) *WNT2*	0.00082	0.011963333	0.37
Intensity FAP (0 - 3) *FN1*	0.00043	0.011963333	0.39
Intensity FAP (0 - 3) *FAP*	0.0000073	0.0005402	0.48

Table S6: Differential gene expression patterns between CRC and their precursor lesions. An FDR-corrected p-value of < 0.05 describes a significant correlation. The confidence interval (CI) indicates whether the genes are highly expressed in CRC (positive) or whether they are down-regulated (negative).

**Gene**	**p-Value**	**FDR adjusted p-Values**	**lower CI**	**higher CI**
PTEN	0.00045	0.006	-732.8	-220.6
SMAD4	0.00019	0.004	-492.5	-172.8
FZD5	0.0022	0.012	-280.1	-66.4
ATG14	0.0021	0.012	-203	-52
NRAS	0.0031	0.015	-192	-44
EGFR	0.00081	0.007	-138	-42
BRAF	0.0099	0.046	-197	-30
WNT3	0.00013	0.003	9	24
FLT4	0.00031	0.005	17	57
PDGFB	0.0016	0.012	27	110
TGFBR1	0.002	0.012	91.6	383.2
FAP	0.0022	0.012	87	387
CCND1	0.00083	0.007	116	394
TGFB1	0.00068	0.007	209	781
VEGFA	2.40E-05	0.001	468	1761
FN1	4.70E-06	0.0003	2487	8173

Table S7: Gene set enrichment analysis of differentially expressed genes based on FAP positivity. Fibroblast positivity (based on FAP expression) correlates with pathways displaying a lower or negative enrichment score (FDR-corrected p-Value < 0,05). Geneset size indicates the number of genes involved in certain pathways, and the leading-edge number represents the number of genes affected by CAF infiltration. UserId is HUGO Gene names of the affected genes.

description	normalized Enrichment Score	pValue	geneset size	leadingEdgeNr.	userId
Insulin signaling pathway	-2.218248174	0	17	11	ARAF;BRAF;KRAS;MAP2K2;MAPK1;MTOR;PIK3R1;RAF1;RPS6KB1;RPTOR;SOS2
Autophagy	-2.318831305	0	18	16	AKT1;ATG14;BECN1;HRAS;KRAS;MAP2K2;MAPK1;MTOR;NRAS;PIK3C3;PIK3R1;PIK3R4;PTEN;RAF1;RPS6KB1;RPTOR
Hepatitis C	-2.027314468	0	16	15	AKT1;ARAF;BRAF;CDKN1A;EGFR;HRAS;KRAS;MAPK1;NRAS;PIK3CA;PIK3R1;RAF1;SOS1;SOS2;TP53
ErbB signaling pathway	-2.027602351	0	22	13	ARAF;BRAF;CDKN1B;EGFR;KRAS;MAP2K2;MAPK1;MTOR;PIK3R1;RAF1;RPS6KB1;SOS1;SOS2
Natural killer cell mediated cytotoxicity	-1.929263525	0.002444988	13	10	ARAF;BRAF;KRAS;MAP2K2;MAPK1;NRAS;PIK3R1;RAF1;SOS1;SOS2
Neurotrophin signaling pathway	-1.878304451	0.014251781	14	13	AKT1;BRAF;HRAS;KRAS;MAP2K2;MAPK1;NRAS;PIK3CA;PIK3R1;RAF1;SOS1;SOS2;TP53
B cell receptor signaling pathway	-1.769902867	0.018018018	12	10	AKT1;HRAS;KRAS;MAP2K2;MAPK1;NRAS;PIK3R1;RAF1;SOS1;SOS2
Fc epsilon RI signaling pathway	-1.769902867	0.018018018	12	10	AKT1;HRAS;KRAS;MAP2K2;MAPK1;NRAS;PIK3R1;RAF1;SOS1;SOS2
Estrogen signaling pathway	-1.74926145	0.016166282	14	11	AKT1;EGFR;HRAS;KRAS;MAP2K2;MAPK1;NRAS;PIK3R1;RAF1;SOS1;SOS2
Sphingolipid signaling pathway	-1.671694848	0.014150943	12	6	KRAS;MAP2K2;MAPK1;PIK3R1;PTEN;RAF1
Hippo signaling pathway	1.38342469	0.101666666	15	8	CCND1;FZD10;FZD2;MYC;TGFB1;WNT1;WNT2;WNT3
Melanogenesis	1.456815046	0.06187291	14	5	FZD10;FZD2;WNT1;WNT2;WNT3
Wnt signaling pathway	1.534000475	0.048657718	11	7	CCND1;FZD10;FZD2;MYC;WNT1;WNT2;WNT3
Human T-cell leukemia virus 1 infection	1.190115595	0.245033112	25	9	CCND1;FZD10;FZD2;MYC;PDGFB;TGFB1;WNT1;WNT2;WNT3
Signaling pathways regulating pluripotency of stem cells	0.878652446	0.622366287	21	5	FZD10;FZD2;WNT1;WNT2;WNT3
Pathways in cancer	0.917481873	0.579288025	52	19	CCND1;CCNE1;CDK4;FGF7;FLT4;FN1;FZD10;FZD2;IGF1;MET;MYC;PDGFB;TGFB1;TGFBR1;VEGFA;VEGFC;WNT1;WNT2;WNT3
Human papillomavirus infection	0.95703373	0.519561815	36	7	FN1;FZD10;FZD2;VEGFA;WNT1;WNT2;WNT3
PI3K-Akt signaling pathway	0.525922657	0.987361768	41	16	CCND1;CCNE1;CDK4;FGF7;FLT1;FLT4;FN1;HGF;IGF1;KDR;MET;MYC;PDGFB;PIK3R5;VEGFA;VEGFC
p53 signaling pathway	1.025798497	0.432907348	12	5	CCND1;CCNE1;CDK1;CDK4;IGF1
Cushing syndrome	1.022306791	0.451199999	20	7	CCND1;CCNE1;FZD10;FZD2;WNT1;WNT2;WNT3

Table S8: Gene set enrichment analysis in FAP-positive fibroblast samples differentiated by low and high FAP expression (%) (FDR-corrected p-Value < 0,05). Geneset size indicates the number of genes involved in certain pathways, and the leading-edge number represents the number of genes affected by the percentage of FAP positive infiltrating CAFs. UserId is HUGO Gene names of the affected genes. A high enrichment score correlates with low FAP expression.

description	normalized Enrichment Score	pValue	size	leadingEdgeNum	userId
Cell cycle	2.060687849	0	17	9	CCNA2;CCND1;CDK1;CDK4;CDKN1A;CDKN2A;MDM2;MYC;TP53
Viral carcinogenesis	1.820922472	0	18	10	CCNA2;CCND1;CDK1;CDK4;CDKN1A;CDKN2A;HRAS;MDM2;NRAS;TP53
Small cell lung cancer	1.518634715	0.072164948	14	5	CCND1;CDK4;FN1;MYC;TP53
Bladder cancer	1.376252411	0.072463768	19	10	CCND1;CDK4;CDKN1A;CDKN2A;EGFR;HRAS;MDM2;MYC;NRAS;TP53
p53 signaling pathway	1.338108497	0.093596059	12	7	CCND1;CDK1;CDK4;CDKN1A;CDKN2A;MDM2;TP53
Thyroid cancer	1.429520126	0.070422535	12	6	CCND1;CDKN1A;HRAS;MYC;NRAS;TP53
MicroRNAs in cancer	1.377879771	0.076923076	29	12	ABCB1;CCND1;CDKN1A;CDKN2A;EGFR;HRAS;MDM2;MET;MYC;NRAS;TP53;WNT3
Cellular senescence	1.279280854	0.11111111	28	10	CCNA2;CCND1;CDK1;CDK4;CDKN1A;CDKN2A;MDM2;MYC;NRAS;TP53
Epstein-Barr virus infection	1.211495306	0.184049079	15	7	CCNA2;CCND1;CDK4;CDKN1A;MDM2;MYC;TP53
Human T-cell leukemia virus 1 infection	1.165925781	0.225490194	25	6	CCND1;CDK4;CDKN2A;MYC;TP53;WNT3
Rap1 signaling pathway	-1.704657678	0.003329634	24	13	AKT1;BRAF;EGF;FGF7;FLT4;HGF;IGF1;PDGFB;PIK3CA;PIK3R1;RAF1;VEGFA;VEGFC
Ras signaling pathway	-1.625504811	0.006644518	26	14	AKT1;EGF;FGF7;FLT4;HGF;IGF1;PDGFB;PIK3CA;PIK3R1;RAF1;SOS1;SOS2;VEGFA;VEGFC
Relaxin signaling pathway	-1.429591112	0.065142857	20	11	ACTA2;AKT1;PIK3CA;PIK3R1;RAF1;SOS1;SOS2;TGFB1;TGFBR2;VEGFA;VEGFC
Chemokine signaling pathway	-1.394916662	0.088779285	13	8	AKT1;BRAF;PIK3CA;PIK3R1;PIK3R5;RAF1;SOS1;SOS2
MAPK signaling pathway	-1.44404783	0.045209903	31	15	AKT1;BRAF;EGF;FGF7;FLT4;HGF;IGF1;PDGFB;RAF1;SOS1;SOS2;TGFB1;TGFBR2;VEGFA;VEGFC
Endometrial cancer	-0.634626975	0.897377422	22	8	AKT1;BRAF;EGF;PIK3CA;PIK3R1;RAF1;SOS1;SOS2
Endocrine resistance	-0.509521765	0.969525958	25	8	AKT1;BRAF;IGF1;PIK3CA;PIK3R1;RAF1;SOS1;SOS2
Non-small cell lung cancer	-0.543105469	0.96158192	23	8	AKT1;BRAF;EGF;PIK3CA;PIK3R1;RAF1;SOS1;SOS2
Kaposi sarcoma-associated herpesvirus infection	-0.66033779	0.891525423	24	8	AKT1;ATG14;PDGFB;PIK3CA;PIK3R1;PIK3R5;RAF1;VEGFA
Human papillomavirus infection	-0.563261658	0.959826275	36	6	EGF;FZD2;RAF1;SOS2;VEGFA;WNT1

Table S9: Gene set enrichment in dependence of high and low FAP staining intensity (FDR-corrected p-Value < 0,05). Geneset size indicates the number of genes involved in certain pathways, and the leading-edge number represents the number of genes affected by the strength of staining. UserId is HUGO Gene names of the affected genes. A high enrichment score correlates with low FAP ex-pression.

description	normalizedEnrichmentScore	pValue	size	leadingEdgeNum	userId
Alcoholism	1.295483762	0.179710144	10	4	ARAF;BRAF;HRAS;SOS2
Insulin signaling pathway	1.586636707	0.028880866	17	13	AKT1;ARAF;BRAF;HRAS;MAP2K2;MTOR;NRAS;PIK3R1;RAF1;RPS6KB1;RPTOR;SOS1;SOS2
Choline metabolism in cancer	1.302049466	0.156934306	17	12	AKT1;EGF;EGFR;HRAS;MAP2K2;MTOR;NRAS;PIK3R1;RAF1;RPS6KB1;SOS1;SOS2
Autophagy	1.642591454	0.015564202	18	14	AKT1;ATG14;BECN1;HRAS;MAP2K2;MTOR;NRAS;PIK3C3;PIK3R1;PIK3R4;PTEN;RAF1;RPS6KB1;RPTOR
Endometrial cancer	1.307174347	0.137440758	22	16	AKT1;ARAF;BRAF;CTNNB1;EGF;EGFR;HRAS;MAP2K2;MYC;NRAS;PIK3R1;PTEN;RAF1;SOS1;SOS2;TP53
Human immunodeficiency virus 1 infection	1.312997347	0.134730539	14	10	AKT1;CDK1;HRAS;MAP2K2;MTOR;NRAS;PIK3R1;RAF1;RPS6KB1;WEE1
Acute myeloid leukemia	1.437251703	0.078947368	18	16	AKT1;ARAF;BRAF;CCND1;HRAS;KRAS;MAP2K2;MAPK1;MTOR;MYC;NRAS;PIK3R1;RAF1;RPS6KB1;SOS1;SOS2
Estrogen signaling pathway	1.342310725	0.11295681	14	10	AKT1;EGFR;HRAS;MAP2K2;NRAS;PIK3R1;RAF1;SOS1;SOS2;TGFA
Neurotrophin signaling pathway	1.318408432	0.154838709	14	10	AKT1;BRAF;HRAS;MAP2K2;NRAS;PIK3R1;RAF1;SOS1;SOS2;TP53
Hepatitis C	1.467101014	0.073170731	16	9	ARAF;BRAF;EGF;EGFR;HRAS;NRAS;PIK3R1;SOS2;TP53
Prostate cancer	-0.408804629	0.99512195	29	4	CCNE1;CDKN1A;IGF1;MDM2
Rap1 signaling pathway	-1.331480256	0.131313131	24	4	FGF7;FLT1;IGF1;VEGFA
PI3K-Akt signaling pathway	-1.267537648	0.151724138	41	11	CCNE1;CDKN1A;FGF7;FLT1;FLT4;FN1;IGF1;MDM2;PDGFB;VEGFA;VEGFC
Pathways in cancer	-1.241189518	0.155133928	52	15	CCNE1;CDKN1A;FGF7;FLT4;FN1;FZD10;IGF1;MDM2;PDGFB;SMAD2;TGFB1;VEGFA;VEGFC;WNT2;WNT3
Ras signaling pathway	-1.269453968	0.181034483	26	4	FGF7;FLT1;IGF1;VEGFA
HIF-1 signaling pathway	-1.152908038	0.291371994	15	4	CDKN1A;FLT1;IGF1;VEGFA
Focal adhesion	-1.145961837	0.294416243	25	4	FLT1;FN1;IGF1;VEGFA
MAPK signaling pathway	-1.162580965	0.295159386	31	5	FGF7;FLT1;IGF1;TGFB1;VEGFA
Small cell lung cancer	-1.144507168	0.332378223	14	3	CCNE1;CDKN1A;FN1
Regulation of actin cytoskeleton	-1.127849105	0.360323886	19	2	FGF7;FN1

Table S10: Gene set enrichment analysis differentiated by adenoma and CRC samples in conjunction with CAFs. Geneset size indicates the number of genes involved in certain pathways, and the lead-ing-edge number represents the number of genes affected by differents between CRC and its pre-cursor lesions. UserId is HUGO Gene names of the affected genes. A high enrichment score corre-lates with low FAP expression.

description	normalized Enrichment Score	pValue	size	leadingEdgeNum	userId
Autophagy	2.513204059	0	18	12	ATG14;BECN1;KRAS;MAPK1;MTOR;NRAS;PIK3C3;PIK3CA;PIK3R1;PIK3R4;PTEN;RPS6KB1
Natural killer cell mediated cytotoxicity	1.963040832	0.008403361	13	8	ARAF;BRAF;KRAS;MAPK1;NRAS;PIK3CA;PIK3R1;SOS2
Insulin signaling pathway	1.880673818	0.023255814	17	10	ARAF;BRAF;KRAS;MAPK1;MTOR;NRAS;PIK3CA;PIK3R1;RPS6KB1;SOS2
Hepatitis C	2.004446534	0	16	11	ARAF;BRAF;EGF;EGFR;KRAS;MAPK1;NRAS;PIK3CA;PIK3R1;SOS2;TP53
Sphingolipid signaling pathway	1.596969297	0.022727273	12	7	KRAS;MAPK1;NRAS;PIK3CA;PIK3R1;PTEN;TP53
GnRH signaling pathway	1.613668525	0.032894737	10	5	EGFR;KRAS;MAPK1;NRAS;SOS2
Alcoholism	1.643122535	0.014388489	10	6	ARAF;BRAF;KRAS;MAPK1;NRAS;SOS2
Neurotrophin signaling pathway	1.671410748	0.01	14	8	BRAF;KRAS;MAPK1;NRAS;PIK3CA;PIK3R1;SOS2;TP53
Apoptosis	1.390169562	0.111888111	11	6	KRAS;MAPK1;NRAS;PIK3CA;PIK3R1;TP53
Estrogen signaling pathway	1.407590573	0.097345132	14	6	EGFR;MAPK1;NRAS;PIK3CA;PIK3R1;SOS2
Glioma	-0.369328686	0.998957246	28	5	CCND1;CDKN2A;IGF1;PDGFB;TGFA
Focal adhesion	-1.214633334	0.198544698	25	5	FLT4;FN1;IGF1;PDGFB;VEGFA
Pathways in cancer	-1.175024319	0.237563452	52	14	CCND1;CDKN2A;FGF7;FLT4;FN1;FZD2;IGF1;MYC;PDGFB;TGFB1;VEGFA;WNT1;WNT2;WNT3
Hippo signaling pathway	-1.21119303	0.241758241	15	9	CCND1;CTNNB1;FZD2;MYC;TGFB1;TGFBR1;WNT1;WNT2;WNT3
Wnt signaling pathway	-1.212144996	0.268208092	11	6	CCND1;FZD2;MYC;WNT1;WNT2;WNT3
Regulation of actin cytoskeleton	-1.182308846	0.274074074	19	3	FGF7;FN1;PDGFB
Rap1 signaling pathway	-1.153635658	0.291886196	24	5	FGF7;FLT4;IGF1;PDGFB;VEGFA
Human T-cell leukemia virus 1 infection	-1.157663441	0.294614572	25	9	CCND1;CDKN2A;FZD2;MYC;PDGFB;TGFB1;WNT1;WNT2;WNT3
Ras signaling pathway	-1.139177666	0.316821465	26	5	FGF7;FLT4;IGF1;PDGFB;VEGFA
Proteoglycans in cancer	-1.110664814	0.341438703	38	10	CCND1;FN1;FZD2;IGF1;MYC;TGFB1;VEGFA;WNT1;WNT2;WNT3
